# Supplementary material for: Survival of immature pre-adult Gnathostoma spinigerum in humans after treatment with albendazole
Source: PLoS One. 2022 Mar 8;17(3):e0264766. doi: 10.1371/journal.pone.0264766 (PMC8903278; doi:10.1371/journal.pone.0264766)
Supplement: Attachment — Submitted filename: PLOS ONE comments reviewer 2.docx [file pone.0264766.s002.docx]

Reviewers’ responses

==============================
ACADEMIC EDITOR:

#Reviewer 2

This paper is very interesting to find the possible clues for the survival of immature pre-adult Gnathostoma spinigerum in humans after treatment with albendazole, addressing the difference in morphology among larval, juveniles, adult and drug-resistant one employing SEM. Although they have important findings, a question is that how can the authors determine due to difference in morphology is the reason for this worm to be drug-resistant? With no molecular assessment, it's very hard to say this kind of worm is drug-resistant. This point should be addressed by the authors.

Response: Thank you and we do agree that more further study is required to prove about drug-resistant *Gnathostoma* worms. Anyway, the survival worms are worth showing evidences of survival albendazole-treated worms and we change the Drug-resistant immature stage (DRIM) to the Survival drug-treated immature stage of *Gnathostoma spinigerum* (STIM). We agree that the DRIM issue requires molecular information of more specimens to be strongly significant confident to confirm this evidence in further work. All responses are already added in the discussion part (lines 309-323).
